# Sand, Sandpaper, and Sandwiches: Evidence From a Masked Compound Priming Task in L1 and L2 Speakers of English

**DOI:** 10.5334/joc.350

**Published:** 2024-02-28

**Authors:** Hasibe Kahraman, Elisabeth Beyersmann

**Affiliations:** 1 School of Psychological Sciences, Macquarie University, Australia; 2 Macquarie University Centre for Reading, Macquarie University, Australia

**Keywords:** compound processing, masked priming, individual differences, bilinguals

## Abstract

This study follows the footsteps of Jonathan Grainger and colleagues by investigating compound processing in English monolinguals and Chinese-English bilinguals using the masked primed lexical decision paradigm. First language (L1) and second language (L2) speakers responded to a semantically transparent compound (e.g., *snowball-SNOW*), a semantically opaque compound (*honeymoon-HONEY*), and an orthographic control condition (e.g., *sandwich-SAND*). Results revealed significantly larger L1 priming effects in transparent and opaque compared to the control condition (Experiment 1A), whereas no significant differences across conditions were observed in L2 speakers (Experiment 1B). We argue that L1 populations are sensitive to morphological structure during the early stages of compound processing, whereas L2 speakers, in particular those with lower levels of language proficiency, employ a form-based type of analysis. Findings are interpreted within the framework of recent monolingual and bilingual models of complex word recognition.

In this article, we present data from masked morphological priming experiments in first-language (L1) and second-language (L2) speakers of English to test differences in the morphological parsing system between the two speaker groups. The question of how readers process morphologically complex words has been debated since the early works of Taft and colleagues (Taft & Forster, 1975). Amongst the most substantial advances in the field are the empirical and theoretical contributions of Jonathan Grainger and his colleagues, which have provided multiple steppingstones on the way to understanding the mechanisms underlying complex word recognition in both monolinguals and bilinguals (e.g., Diependaele et al., 2011; Giraudo & Grainger, 2000, 2003b; Grainger et al., 1991). Over the past 30 years, several key milestones have been reached in the development of methodological tools and theoretical frameworks of complex word processing, on which Jonathan Grainger had a remarkable impact. Within the context of this special issue, we honour several of Grainger’s most notable contributions to the field of complex word recognition, which have become and continue to be indispensable in recent streams of reading research, including the current study.

The primary focus of the here presented series of masked priming experiments was to provide insights into the morphological parsing system in bilinguals, by carefully examining similarities and differences between masked morphological priming effects in L1 and L2. First, we begin with a summary of what is best understood due to more extensive experimentation in this area in past years: morphological processing in L1. Then we move on to discussing differences and similarities between morphological processing in L1 and L2, which, as will be outlined below, is less well understood.

## Masked Morphological Priming in L1

One of most significant methodological advances in the study of morphological processes in L1 readers has been the groundwork by Jonathan Grainger and colleagues who introduced masked priming to the field of complex visual word recognition (Giraudo & Grainger, 2000, 2003b; Grainger et al., 1991). This work formed the basis for a surge in masked morphological priming studies (e.g., Beyersmann et al., 2018; Grainger et al., 1991; Kahraman & Kırkıcı, 2021; Viviani & Crepaldi, 2022), and within only a few years, masked priming became what is now known to be the gold standard for the investigation of morphological processes in reading (for reviews, see Amenta & Crepaldi, 2012; Marelli et al., 2020; Rastle & Davis, 2008).

Within the years that followed, it was shown that target words preceded by semantically transparent affixed words (e.g., *farmer-FARM*) yield facilitation that is comparable to targets preceded by semantically opaque affixed words (e.g., *corner-CORN*; Longtin et al., 2003; Rastle et al., 2004). Semantically *transparent* prime-target pairs were defined as those with semantic prime-target overlap, whereas semantically *opaque* items were selected such that there was a shared morphological but not a shared semantic relationship between the prime and the target (e.g., the embedded word *corn* is not semantically related to the meaning of *corner)*. Critically, when compared against a monomorphemic control condition (*cashew-CASH*), it was found that both the semantically transparent and opaque conditions yielded stronger priming relative to the control, suggesting that the observed priming effects were not just due to orthographic prime-target overlap (e.g., Beyersmann et al., 2016; Marslen-Wilson et al., 2008; Rastle & Davis, 2008). This widely replicated pattern of results neatly showed that under masked priming conditions, readers are sensitive to a semantically independent form of morphological structure, suggesting that morphological segmentation likely takes place within the early, pre-lexical stages of visual word recognition, thereby challenging supra-lexical account of morphological processing by which morphological information is not processed until the post-lexical stages of word recognition (Giraudo & Grainger, 2001, 2003a).

Over the years, masked morphological priming effects in L1 speakers have been interpreted within a wide range of theoretical models. Current theories can be broadly divided into the decompositional view of morphological processing (for a recent review, see Stevens & Plaut, 2022), by which the segmentation of complex words is based on the activation of explicit morphemic units, and the distributional view of morphological processing, which assumes that morphology is a by-product of mappings between orthography and semantics (e.g., Baayen et al., 2011; Feldman et al., 2002, 2004; Marelli et al., 2020; Plaut & Gonnerman, 2000). Within the decompositional stream, several influential models of complex word processing have been developed over the past decade, including obligatory decomposition accounts (e.g., Taft, 2003; Taft & Ardasinski, 2006; see also Taft, 2023, for an updated version of this account), Giraudo and Grainger’s supra-lexical account (e.g., Giraudo & Grainger, 2001, 2003a) and dual-route accounts, where the latter propose that complex words simultaneously activate orthographic whole-word representations as well as their embedded morphemic subunits (e.g., Beyersmann, Coltheart, et al., 2012; Diependaele et al., 2009; Kuperman et al., 2009; Schreuder & Baayen, 1995). Two of Grainger’s most recent theoretical frameworks fall under this umbrella: Grainger and Ziegler’s (2011) dual-route model of orthographic processing and Grainger and Beyersmann’s word and affix model (Beyersmann & Grainger, 2023; Grainger & Beyersmann, 2017). Given that the current study was concerned with the direct comparison between masked morphological priming effects in L1 and L2 speakers, it is important to underline the notion that, despite obvious discrepancies between the distributional and decompositional accounts of morphological processing, all models agree that L1 speakers rapidly and automatically extract morphemic information from print.

## Masked Morphological Priming in L2

What is known with regards to morphological processing in L2 is less clear. There has been an increase in studies that have investigated if and how the mechanisms of complex word recognition in L2 differ from complex word processing in the L1. Once again, Jonathan Grainger and his colleagues were amongst the first to use the masked priming paradigm to study the automaticity of visual word recognition processes in bilinguals (e.g., Bijeljac-Babic et al., 1997; Chauncey et al., 2008; Grainger, 1998; Grainger & Dijkstra, 1992). This work not only helped bridge the gap between the L1 and L2 reading literature, but also provided an important basis for the investigation of morphological processing effects in bilinguals.

The results from masked morphological priming research in L1 and L2 speakers have revealed conflicting findings. Some studies have found that L1 and L2 morphological priming is comparable (e.g., González Alonso et al., 2016; Kahraman, 2022), while others have shown that L2 speakers are more reliant on orthographic form processing (Diependaele et al., 2011; Heyer & Clahsen, 2015; J. Li et al., 2017; J. Li & Taft, 2020; M. Li et al., 2017; Viviani & Crepaldi, 2022; for a detailed summary of the evidence, see Table 1 in Kahraman & Beyersmann, 2023). For example, Heyer and Clahsen (2015) investigated the sensitivity of L2 users of English to complex word structures in English. They reported that lexical decisions of L2 users of English were enhanced by a previous presentation of a prime that had an apparent morphological relationship (e.g., *hunter, corner*) to the target word (e.g., *HUNT, CORN*) as well as that of a prime that had a purely orthographic relationship (e.g., *freeze*) to the target (e.g., *FREE*), as opposed to L1 English speakers who only showed priming in the two morphological conditions (also see M. Li et al., 2017). In addition to the investigation of derivational suffixes, other studies have presented corroborating evidence in favour of bilinguals’ sensitivity to the orthographic structure using derivational prefixes. For example, in a masked primed lexical decision task with Chinese-English bilinguals, J. Li and Taft (2020)^1^ showed that L2 prime-target pairs that had a transparent (e.g., *disagree*-*AGREE*), opaque (e.g., *mischief*-*CHIEF*) and form (e.g., *stranger*-*ANGER*) relationship that all significantly speeded up the reaction times.

**Table 1:** Participant Demographics.


VARIABLE		M	max	M	max
	
		L1 GROUP (*n* = 95)		L2 GROUP (*n* = 112)	

**Years** 2	of education	**13.04**		**15.93**	

	Spent in an Eng.-speaking country	**20.06**		**6.34**	

**Age of**	First contact with Eng.^3^	**0.92**		**8.63**	

	First reading Eng.^4^	**4.24**		**10.96**	

	Fluent reading Eng.^5^	**7.02**		**18.17**	

**Level of Proficiency in** 6	Speaking Eng.	**9.59**	10	**7.08**	10

	Understanding spoken Eng.	**9.69**	10	**7.35**	10

	Reading in Eng.	**9.49**	10	**7.25**	10

**Current exposure to Eng. in** 7	Interacting with friends	**9.59**	10	**6.27**	10

	Interacting with family	**9.48**	10	**2.09**	10

	Watching TV	**8.55**	10	**6.06**	10

	Listening to radio/music	**8.21**	10	**5.99**	10

	Reading	**9.26**	10	**7.73**	10

	Language/Lab instruction	**6.06**	10	**5.02**	10

**Eng**.	AoA^8^	**4.49**		**17.43**	10

	Accentedness^9^	**0.91**	10	**4.39**	10

	Nonnativeness^10^	**0.76**	10	**5.71**	10


*Note*: Eng = English; AoA = Age of Acquisition.

As the above summary shows, the primary focus of previous work in bilinguals has been on the processing of affixed words (e.g., Diependaele et al., 2011, but see Table 1 in Kahraman & Beyersmann, 2023 for a comprehensive list of studies). Much less is known regarding the processing of compound words in L2 speakers. In the current study, we aimed to shed new light on the question of how morphologically complex words are processed in the L2 reading system by examining the compound processing mechanisms. Compound words (e.g., *farmhouse*) are composed of two embedded stem constituents that typically exist as free-standing words (*farm* and *house*). The stem of affixed words determines the primary meaning of the word, with the affix modifying the stem’s meaning. Therefore, it is possible that the processing and segmentation of affixed words into morphemes during reading requires more language expertise than the decomposition of compound words.

Studies with developing readers provide some initial evidence for differences in the developmental time-course by which compound word processing and affixed word processing are acquired by children. For example, a different pattern of masked morphological priming effects has been reported depending on whether the target words are preceded by compound words (e.g., *farmhouse-FARM*) or affixed words (e.g., *farmer-FARM*). With compound word primes, English speaking primary school children and adults produce significant morpho-semantic (e.g., *farmhouse-FARM*) and morpho-orthographic (e.g., *butterfly-BUTTER*) priming effects, which are both significantly larger than form priming effects, suggesting that children as young as Year 3 are already proficient at segmenting compound words into morpho-orthographic subunits (Beyersmann et al., 2019). In contrast, affixed word priming studies with children have reported priming with transparent (e.g., *farmer-FARM*) but not with opaque affixed words (e.g., *corner-CORN*), indicating that the automaticity of affix segmentation mechanisms is a much later acquired milestone (e.g., Beyersmann, Castles, et al., 2012; Dawson et al., 2018, 2021). Given that developing readers showcase a different developmental time-course regarding the acquisition of compound compared to affix segmentation mechanisms, it is possible that L2 speakers undergo a similar learning trajectory by which compound words and affixed words are handled in different ways.

## The Present Study

The aim of the present study was to directly compare the mechanisms and automaticity of compound word processing in L1 and L2 speakers of English. To our knowledge, there are only three prior studies that have directly investigated compound word processing using masked priming in bilinguals comparing L1 and L2 access to morphological information (González Alonso et al., 2016; M. Li et al., 2017; Uygun & Gürel, 2017), but the results are not conclusive. González Alonso et al. (2016) examined L1 and L2 speakers of English whose L1 is German using masked primed lexical decision task (LDT). Significant priming effects were reported for both groups in the two transparent morphological conditions (e.g., *fund-FUNDRAISER; raiser-FUNDRAISER*) but not in the orthographic control conditions (e.g., *funk-FUNDRAISER; raisin-FUNDRAISER*), relative to an unrelated condition (e.g., *cool-FUNDRAISER*). Note that complex words were presented as visible targets in this study (*fund*-*FUNDRAISER*), rather than as masked primes (*fundraiser-FUND*). Therefore, complex word processing was open to strategic factors in this task. Moreover, their orthographic condition did not include an orthographic monomorphemic control with an embedded word as typically used in compound studies (e.g., *sandwich-SAND*).

A similar pattern of results has been reported by M. Li et al., (2017), who conducted a masked primed LDT in L1 English monolinguals and L1 Chinese – L2 English bilinguals, using an additional opaque compound condition. Both participant groups showed comparable facilitation effects in the transparent (e.g., *toothbrush-TOOTH; toothbrush-BRUSH*) and opaque (e.g., *honeymoon-HONEY; honeymoon-MOON*) compound conditions, independently of semantic transparency (i.e., priming occurred for both transparent and opaque conditions) and position (i.e., priming occurred for both initial and final constituents). Interestingly, as opposed to the earlier findings by González Alonso et al. (2016), M. Li et al. (2017) found that the L2 group showed significant priming when form overlap occurred in initial position (e.g., *restaurant-REST*), but priming was not significant when form overlap occurred in the word final position (e.g., *tomorrow-ROW*). The authors took this evidence to suggest that orthographic priming is constrained by position, whereas morphological priming is not. However, form effects need to be interpreted cautiously because they were obtained from comparatively fewer item numbers in this condition. Also, the authors employed a longer priming duration in L2 speakers, making the lexical decision task relatively vulnerable to strategic reading mechanisms. Using a similar prime-target manipulation with compound targets in L1 English speakers and L1 Turkish – L2 English speakers, Uygun and Gürel (2017) found that, in both groups, the transparent condition (*head-HEADACHE; ache-HEADACHE*) and the partially opaque condition (*e.g., grape-GRAPEFRUIT; fruit-GRAPEFRUIT*) produced significant priming effects, while the form condition (e.g., *croco-CROCODILE; dile-CROCODILE*) did not. However, given that the form condition did not use a real word prime (*croco*), it is unclear if the observed effects are lexical rather than morphological in nature. Thus, questions remain regarding the nature and automaticity of compound word processing in L2 learners.

The present study addressed this gap by examining early automatic processing of English compound words in L1 English monolinguals and L1 Chinese – L2 speakers within a series of two masked primed lexical decision experiments. Following earlier findings (Beyersmann et al., 2019; Fiorentino & Fund-Reznicek, 2009), we hypothesised that L1 speakers of English should show masked priming effects in transparent compound (e.g., *snowball*-*SNOW*) and opaque compound (e.g., *butterfly*-*BUTTER*) conditions but not in the form condition (e.g., *sandwich*-*SAND*). Concerning the L2 English bilinguals, we specified three different hypotheses: (1) If L2 speakers decompose compound words in the same way as L1 speakers (e.g., González Alonso et al., 2016), we would expect to replicate the pattern of morphological priming seen with L1 speakers (i.e., significant priming in the transparent and opaque compound conditions, but not in the form condition). (2) If it is true that form priming is more prominent in L2 speakers (e.g., J. Li et al., 2017), we would expect to see significant priming in all three priming conditions. (3) If L2 speakers are more reliant on whole-word processing than morphological decomposition (e.g., Clahsen et al., 2010; Giraudo & Grainger, 2001), whole-word access would be expected to have priority over morphological decomposition, and hence resulting in significant priming in the transparent priming condition, but not in the opaque compound and form conditions, both of which were expected to yield comparable priming effects.

Finally, all participants were asked to complete spelling, vocabulary, and reading tests to examine the hypothesis that individual differences in vocabulary and lexical expertise modulate morphological processing (e.g., Andrews & Hersch, 2010; Andrews & Lo, 2013; Beyersmann et al., 2015; Kahraman & Kırkıcı, 2021). We preregistered these predictions along with the method, procedure, and data analysis plans (https://osf.io/k675y).

## Experiment 1A: L1 English Speakers

Experiment 1A served as a replication of Beyersmann et al. (2019).

### Method

#### Participants

Hundred twenty-six L1 speakers of English (mean age: 20.99, range: 17–60, 91 females) with normal or corrected-to-normal vision and no history of neurological disorders completed the first experiment for course credit. Sixty-five L1 speakers completed the experiment online (using webDMDX, Forster & Forster, 2003; Witzel et al., 2013), whereas 61 completed the experiment in person (using DMDX, Forster & Forster, 2003). The in-person experiments were planned and pre-registered before the COVID-19 pandemic. With the beginning of the COVID-19 pandemic, all in-person participant testing was put on hold and therefore temporarily shifted to an experimental platform online, which resulted in a split between the in-person and online testing modality. All participants were undergraduate students in the department of Psychology at Macquarie University, Sydney, Australia and completed the Language Experience and Proficiency Questionnaire (LEAP-Q; Marian et al., 2007). Thirty-one participants who reported speaking and/or understanding a language other than English fluently before the age of 10 were omitted prior to statistical analyses (mean age: 21.45, range: 17–60, 68 females). Participant demographics are presented in Table 1.

#### Materials

Ninety-six monomorphemic words from Beyersmann et al. (2019) served as word targets. These were preceded by 32 transparent compounds (*snowball*), 32 opaque compounds (*butterfly*), and 32 form word primes (*sandwich*; see Table 2 for examples of stimuli in each condition as well as word-level characteristics). Target words in the three morphological conditions were originally extracted from the Children’s Printed Word Database and matched on their word frequency, number of letters, number of phonemes, and number of syllables, orthographic neighbourhood, and phonological neighbourhood (given that the items were taken from materials developed for primary school children; for descriptive statistics from the Children’s Printed Word Database, see Table 1 in Beyersmann et al., 2019). Given that participants in the current study were adults, word-level characteristics were additionally extracted from the SUBTLEX_UK database (see Table 2; Van Heuven et al., 2014). Target words in the three compound type conditions were matched on word frequency (Zipf values), orthographic neighbourhood, and phonological neighbourhood. However, prime words were not matched on their word frequency (Zipf values), orthographic neighbourhood, or phonological neighbourhood across the three prime type conditions and therefore included as covariates in our analyses.

**Table 2:** Examples For Prime–Target Pairs Across Conditions and Word-Level Characteristics.


	TRANSPARENT			OPAQUE			FORM		
		
	REL	UNREL	TARGET	REL	UNREL	TARGET	REL	UNREL	TARGET
		
	*snowball*	*passport*	*SNOW*	*butterfly*	*household*	*BUTTER*	*sandwich*	*vampire*	*SAND*

**Word Freq**	3.21 (0.54)	3.42 (0.94)	4.76 (0.52)	3.32 (0.71)	3.38 (0.73)	4.51 (0.65)	4.02 (0.5)	3.69 (0.92)	4.83 (0.67)

**OrthN**	0.03 (0.18)	0.13 (0.42)	8.34 (4.93)	0.19 (0.4)	0.59 (1.32)	8.38 (4.88)	0.22 (0.49)	0.16 (0.45)	10.75 (6.11)

**PhonN**	0.65 (0.56)	1.04 (1.2)	16.34 (8.17)	0.65 (0.71)	1.26 (2.94)	16.56 (7.40)	2.10 (1.97)	1.19 (1.5)	19.84 (9.05)


*Note*: Rel = Related Prime; Unrel = Unrelated Prime; Word Freq = Word Frequency; OrthN = Orthographic Neighbourhood; PhonN = Phonological Neighbourhood.

For the purpose of the lexical decision task, 96 nonword targets were created by substituting one letter in a word (*door–dror*). Half of the primes for nonword targets comprised complex words, and the other half of the primes were monomorphemic words that were preceded by either a related or an unrelated word prime (*bookshelf–BOLK* vs. *dishwater–DROR*). Word and nonword targets were matched on length. The stimuli were divided into two counterbalanced lists so that each target appeared only once in each list in a different priming condition.

#### Procedure

The in-person experiment was administered using the DMDX software package (Forster & Forster, 2003). All stimuli were centrally presented in Courier New font. Each trial began with the presentation of a 500-msec forward mask of hash keys, followed by a 50-msec prime in lowercase and then the target. The target remained present until response or until three seconds had elapsed.

The online version of the experiment used the same procedure, except that the experiment was delivered using webDMDX, a web-deliverable implementation of DMDX (Forster & Forster, 2003; Witzel et al., 2013). For this, a self-extracting .zip file was formed with three essential applications: the .rtf script to control the experiment, the .bat file to run the experiment, and the poster.exe file to send off data over the web to a data repository at the University of Arizona. This file was then shared with participants. WebDMDX allowed participants to temporarily download the experiment on their computer and run the experiment locally. For display timing, WebDMDX relies on the Windows operating system of the host computer to report its refresh rate of monitor,^11^ and it then calculates the number of refresh intervals for the closest approximation to the specified duration. The viability of webDMDX for psycholinguistic research has been tested, and it was suggested as a reliable tool for masked repetition priming experiments that require short and tightly controlled display durations (e.g., Witzel et al., 2013; Woods et al., 2015).

After participants completed the lexical decision task, a test battery composed of spelling, vocabulary, and reading tests were administered, which we describe below in detail (see Appendix B for items used in each test at https://osf.io/cqkw3/?view_only=688e8ce2d84b4088b357e7ba1a5b4192).

### Measures of Individual Differences for Lab-Testing

#### Spelling Test

Individual differences in spelling proficiency were assessed based on participants’ performance in a spelling dictation test and a spelling recognition test. Items in both subtests were taken from Kahraman and Kırkıcı (2021). In the spelling dictation test, participants were asked to listen to individual words on the recording and to spell the words they heard. They heard 30 words, and a sentence for each with the word in it. The score was calculated by counting the total number of words spelled correctly. In the spelling recognition test, participants were asked to select the correctly spelled option in a list of 30 items. The score was calculated based on the number of correct answers. Individual spelling proficiency scores were obtained by summing the scores of the spelling dictation and spelling recognition tests.

#### Vocabulary Test

The vocabulary test was productive in nature and measured if participants could provide target words in a specific context. The test was adapted from Kahraman and Kırkıcı (2021) and included 16 target words (e.g., essential). Participants were provided the first and last letter as a clue (e.g., E_______L). In addition, participants were given a list of words that typically co-occur with the target word (e.g., essential characteristic, essential component, etc.). A sentence was also provided with each target word, which each included a gap in the position of the target word, such that participants had additional context to come up with the target.

#### Reading Fluency Test

In the reading fluency test, participants read a text in one minute. In intervals of approximately 60 words, participants selected one out of three words coherent with the passage or indicated if a given statement was true or false. Items were taken from Kahraman and Kırkıcı (2021), and scores were calculated by counting the number of words read and subtracting 50 words for every incorrect selection.

### Measures of Individual Differences for Online Testing

#### Spelling Test

For the spelling test, participants were asked to select the correctly spelled option in a list of 60 items. Items were adapted from Kahraman and Kırkıcı (2021), and the score was the number of correct responses^12^.

#### Vocabulary Test

In the vocabulary knowledge test, participants were presented with individual words in uppercase letters and asked to select the option that best corresponded to the one in uppercase. This is the receptive version of productive vocabulary knowledge test used in previous experiment that allowed us to successfully embed test items within webDMDX. There were 60 questions in the test, and the score was the number of correct responses.

#### Reading Comprehension Test

In the reading comprehension test, participants were instructed to read 10 incomplete sentences and to select the answer that best completed each sentence. The test used in the previous experiment required participants to read only for a limited time (a minute), which is hard to control in webDMDX. Additionally, it required selecting the correct option at the end of each paragraph and moving on the next paragraph, which is beyond what we could achieve with webDMDX. Hence, in the online version of the test, we generated the questions, and the score was the number of correct responses.

### Results and Discussion

Lexical decisions to word targets were analysed as follows. Incorrect responses were removed from the reaction time (RT) analyses. One participant was removed since their mean nonword response accuracy was lower than chance level (50%). Latencies below 300 or above 3000 msec were considered as extreme values and removed (17 datapoints). Data points whose standardised residuals were larger than 2.5 in absolute value (Baayen, 2008) were removed (2.5 % of all data). Mean RTs and standard deviations (SDs) are presented in Table 3.

**Table 3:** Mean Lexical Decision Times (Msec) and Error Rates across L1 English Participants (SD).


(*n* = *94*)	TRANSPARENT		OPAQUE		FORM	
		
	RELATED	UNRELATED	RELATED	UNRELATED	RELATED	UNRELATED

Mean	566 (112)	601 (114)	598 (134)	627 (129)	611 (132)	632 (139)

Error Rates	0.03 (0.18)	0.04 (0.19)	0.05 (0.21)	0.05 (0.23)	0.06 (0.24)	0.06 (0.25)

**Effect Size**	35*		29*		21*	


*Note*: An asterisk (*) indicates statistical significance.

Correct response latencies and accuracies for word targets were analysed with linear mixed-effects (LME) models with participants and items as crossed random variables. LME models were implemented in the lme4 package (Version 1.1–33; Bates et al., 2014) and lmerTest package (Version 3.1–3; Kuznetsova et al., 2017) in the statistical software R (Version 4.2.3; R Development Core Team, 2016). A boxcox power transformation of the RTs indicated all RTs be inverse-transformed (i.e., –1000/RT) to reduce the positive skew in the distributions. The significance of the fixed effects was determined with Type III model comparisons using the anova Type 3 function in the ‘car’ package (Version 3.1-2; Fox & Weisberg, 2019). To assess whether the effect of testing modality (lab-based or web-delivered) influenced priming results, we included ‘testing modality’ as a factor in our analyses. The final model included three fixed effects factors (relatedness: related, unrelated; prime type: transparent, opaque, form; testing modality: in-person, web-delivered), their interactions, and two random effects factors (random intercepts for subjects and items). Standardised trial order was included into the model to control for effects of fatigue or habituation. Standardised prime frequency, orthographic neighbourhood, and phonological neighbourhood were also added as covariates. A reduced random slope structure (Barr et al., 2013) was used to avoid Type I error.^13^ The pairwise comparisons between significant main and/or interaction effects followed the application of LME model using the emmeans package (Lenth, 2023) in R. For the proficiency test analyses, we added each standardised test score to the main LME model as interactions one at a time to analyse whether language proficiency tests or variables from LEAP-Q^14^ contributed significantly to producing a better fitting model. The proficiency model included Relatedness (Related vs. Unrelated), Prime Type (Transparent vs. Opaque vs Form), and standardised test scores as fixed factors and their interactions.^15^ The model also included subjects and items as crossed random effect variables.

The RT analyses yielded a highly robust main effect of prime type (χ^2^(2) = 20.97, *p* < .0001) that significantly interacted with relatedness (χ^2^(2) = 9.83, *p* < .001), due to smaller priming in the form-related condition than the transparent and opaque conditions. Three-way interaction between relatedness, prime type, and testing modality was not significant (χ*^2^*(2) = 1.41, *p* = .49), showing that testing modality did not moderate priming effects in the transparent, opaque, and form conditions. Trial order was a significant covariate (χ*^2^*(1) = 4.99, *p* = .02). None of the other covariates were significant (χ^2^(1) = 0.06, *p* = .79 for prime frequency; χ^2^(1) = 1.64, *p* = .19 for orthographic neighbourhood; χ^2^(1) = 0.18, *p* = .67 for phonological neighbourhood).

Post-hoc tests were run to compute contrasts between conditions using the emmeans package (Lenth, 2023). Each prime type was compared against their corresponding unrelated control condition, revealing that priming effects were significant in transparent, opaque, and form conditions (see top three rows in Table 4).

**Table 4:** LME Results for L1 English.


CONTRAST	estimate	SE	z.ratio	p.value

unrelated-related Form	0.06	0.02	3.73	0.0002

unrelated-related Opaque	0.10	0.02	6.77	<.0001

unrelated-related Transparent	0.12	0.01	8.22	<.0001

Interaction Form*Opaque	–0.05	0.02	–2.22	0.03

Interaction Opaque*Transparent	–0.02	0.02	–0.84	0.40

Interaction Form*Transparent	–0.06	0.02	–3.05	0.002


*Note*: Results are averaged over the levels of factor modality.

The interaction between relatedness and prime type was decomposed into three individual contrasts (see bottom three rows in Table 4) to determine the contribution of (a) morpho-orthographic processing and (b) morpho-semantic processing. The first contrast was used to explore the influence of morpho-orthographic processing on masked priming by comparing priming in the opaque condition against priming in the form condition. The last two contrasts were used to examine the influence of morpho-semantics by comparing priming in the transparent condition against priming in the opaque and form conditions. Importantly, the interaction between transparent and opaque priming was not significant (*β* = –0.02, SE = 0.02, z = –0.84), confirming the similar magnitude of priming for the transparent and opaque conditions. The interaction between form and transparent condition (*β* = –0.06, SE = 0.02, z = –3.05) and between opaque and form primes (*β* = –0.05, SE = 0.02, z = –2.22), was significant, due to greater priming in the opaque compound condition than in the form condition and greater priming in the transparent compound condition than in the form condition.

None of the proficiency tests^16^ or individual variables significantly interacted with relatedness and prime type (χ^2^(2) = 0.51, *p* = 0.77 for spelling, χ^2^(2) = 0.12, *p* = 0.94 for vocabulary, χ^2^(2) = 0.12, *p* = 0.94 for reading, χ^2^(2) = 0.08, *p* = 0.95 for the composite score of language proficiency and exposure).^17^

The results are consistent with Beyersmann et al. (2019)’s earlier findings and thus provide confirmative evidence for the automaticity of compound word processing in monolinguals. The only difference between the current results and Beyersmann et al.’s was the significant form priming effects herein reported. The significantly larger priming effects in the transparent and opaque conditions relative to the form condition suggest that L1 speakers rapidly decompose compound words into morphemic constituents, independently of semantics. Given that the masked compound primes were displayed so briefly (i.e., 50 msec) that participants were unaware of their existence, it can be concluded that morphological processing occurred during the early, automatic stages of lexical processing.

## Experiment 1B: L2 Speakers of English

A set of pre-registered analyses was conducted to test the priming effects in L2 English speakers and to directly compare the effects sizes in L1 and L2.

### Method

#### Participants

Hundred twenty-five L2 speakers of English (mean age: 26.1, range: 17–63, 84 females) with normal or corrected-to-normal vision and no history of neurological disorders attended the second experiment for course credit or for financial reimbursement. Sixty-eight L2 speakers completed the experiment online (using webDMDX, Forster & Forster, 2003; Witzel et al., 2013), whereas 57 completed the experiment in person (using DMDX, Forster & Forster, 2003). The participants comprised both graduate and undergraduate students from Macquarie University, as well as individuals residing in Sydney. According to the LEAP-Q responses, 112 (mean age: 26.1, range: 17–54, 74 females) L2 speakers of English reported Chinese as their native language, and it was the dominant language for the majority of speakers. The mean length of formal education they received was 15.93 years (*SD* = 2.81). The mean age when participants began acquiring English was 8.63 (*SD* = 4.22), and the age when they became fluent in English was 17.43 years (SD = 6.82). The mean level of proficiency on a rating scale from 0–10 in speaking, understanding, and reading in English was 7.08 (*SD* = 1.58), 7.35 (*SD* = 1.52) and 7.25 (*SD* = 1.65), respectively. Participant responses to the rest of the questions are presented in Table 1.

### Materials and Procedure

As in Experiment 1.

### Results and Discussion

The data were cleaned and analysed in the same way as for Experiment 1. Five participants were removed since their nonword response accuracies were below chance level (50 %). Mean RTs and standard deviations (SDs) are presented in Table 5. The RT analysis using a reduced random effect structure for the L2 group only yielded a robust significant main effect of relatedness (χ2(1) = 88.53, *p* < .0001), due to smaller reaction times for targets followed by related primes. Unlike in the Experiment 1A, the interaction of relatedness with prime type was not significant (χ2(2) = 3.55, *p* = 0.17), demonstrating similar magnitudes of priming for the transparent, opaque, and form conditions. Trial order and number of phonological neighbourhood were significant covariates (χ*^2^*(1) = 6.22, *p* < .01, χ*^2^*(1) = 4.23, *p* = .04, respectively). The main effect of testing modality was not significant (χ^2^(2) = 2.92, *p* = 0.09) that showed a lack of three-way interaction with relatedness and prime type (χ^2^(2) = 0.39, *p* = 0.83), demonstrating priming effects in transparent, opaque, and form condition were not moderated by whether experiments were conducted in-person or online (see Table A1 in Appendix A at https://osf.io/cqkw3/?view_only=688e8ce2d84b4088b357e7ba1a5b4192). Due to this non-significant three-way interaction, we did not conduct further post-hoc tests.

**Table 5:** Mean Lexical Decision Times (Msec) and Error Rates across L2 English Participants (SD).


(*n* = 105)	TRANSPARENT		OPAQUE		FORM	
		
	RELATED	UNRELATED	RELATED	UNRELATED	RELATED	UNRELATED

Mean	706 (241)	744 (248)	735 (256)	773 (259)	733 (235)	769 (254)

Error Rates	0.04 (0.20)	0.04 (0.20)	0.07 (0.27)	0.07 (0.27)	0.06 (0.23)	0.07 (0.25)

**Effect Size**	38*		38*		36*	


*Note*: An asterisk (*) indicates statistical significance.

None of the proficiency tests significantly interacted with relatedness and prime type (χ^2^(2) = 3.32, *p* = 0.19 for spelling, χ^2^(2) = 3.37, *p* = 0.18 for vocabulary, χ^2^(2) = 3.38, *p* = 0.18 for reading tests). However, the composite score of English language proficiency/exposure yielded a marginal three-way interaction with relatedness and prime type, χ^2^(2) = 5.68, *p* = 0.05, due to lower form priming and higher opaque priming in participants with high composite scores.

**Figure 1:**
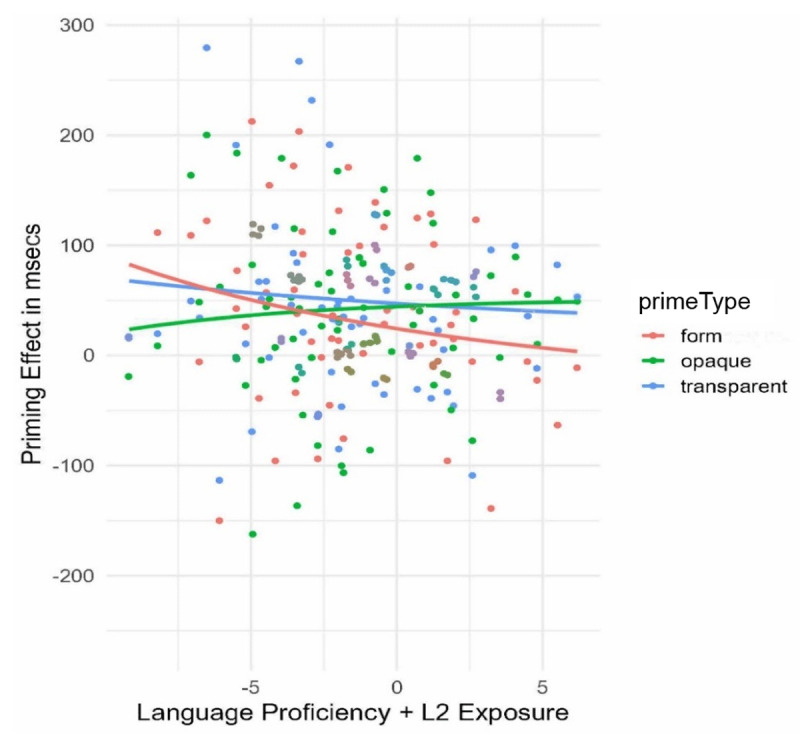
Form Priming as a Function of Language Proficiency/Exposure in L2 English. *Note*: The “observed values” that make up the scatterplot are provided in raw RT scores, but the models are fitting “inverse RT”, which results in curves instead of straight lines.

As seen in Figure 1, priming effects obtained from the form condition decreased with increasing proficiency, while opaque priming increased. In the high spectrum of the proficiency distribution, form priming effects were entirely absent (*β* = 0.02, SE = 0.02, z = 0.71, *p* = .47), whereas opaque priming became robust (*β* = 0.11, SE = 0.02, z = 4.21, *p* < .0001), demonstrating a more L1-like priming pattern. Participants with lower levels of scores, however, seem to be more expert at identifying embedded form units independently of morphemic structure.

### Combined L1 and L2 Analysis

In order to more directly compare differences in L1 and L2 morphological priming, the interaction of relatedness (related vs unrelated), prime type (transparent vs opaque vs form), group (L1 vs L2), and testing modality (in-person, web-delivered) was assessed in the combined analysis of Experiments 1A and 1B. The three-way interaction between the fixed effects of relatedness, prime type, and group was not significant^18^ (χ^2^(2) = 2.69, *p* = .26, nor was the four-way interaction between relatedness, prime type, group, and testing modality (χ^2^(2) = 1.31, *p* = .52 see Table A2 in Appendix A for the full model output at https://osf.io/cqkw3/?view_only=688e8ce2d84b4088b357e7ba1a5b4192).

The pairwise contrasts between the levels of each prime type showed that none of the interactions were significant (see bottom three rows of Table 6). Overall, the results of Experiments 1A and 1B provide evidence for orthographic (form) priming in both L1 and L2 English groups, as evidenced by significant priming effects in the transparent, opaque and form conditions. In addition, when analysed separately, only the L1 group showed clear evidence for additional facilitation in the two morphological condition relative to the orthographic control.

**Table 6:** Post-hoc Contrasts between the Levels of Each Prime Type across L1 and L2 Groups.


GROUP	CONTRAST	ESTIMATE	SE	Z.RATIO	P.VALUE

**L1**	RE.Form	0.06	0.01	4.00	<.0001

	RE.Opq	0.10	0.01	7.34	<.0001

	RE.Trnsp	0.12	0.01	9.06	<.0001

**L2**	RE.Form	0.06	0.01	4.58	<.0001

	RE.Opq	0.08	0.01	6.41	<.0001

	RE.Trnsp	0.09	0.01	7.19	<.0001

**L1 vs L2**	RE.Form_Opq	–0.02	0.02	–0.98	0.33

	RE.Form_Trnsp	–0.03	0.02	–1.63	0.10

	RE.Trnsp_Opq	0.01	0.02	0.63	0.53

	RE.Trnsp	0.03	0.02	1.80	0.07

	RE.Opq	0.01	0.02	0.91	0.36

	RE.Form	–0.01	0.02	–0.40	0.69


*Note*: RE = relatedness effect; Form, Opq, Trnsp = levels of prime type. ^a^Results are averaged over the levels of factor modality.

## General Discussion

The current study investigated the early influences of semantics, morphology, and orthography on compound processing in both L1 and L2 English speakers within two masked primed lexical decision experiments. The aim was to replicate and extend the previous investigation of pseudo-compound priming effects in L1 speakers (Beyersmann et al., 2019; Fiorentino & Fund-Reznicek, 2009) to second language users of English and to test for the effects of individual differences in language proficiency on morpho-orthographic and morpho-semantic priming in both L1 and L2 speakers.

The results of Experiment 1A provided evidence for comparable transparent and opaque priming effects in L1 speakers, replicating Beyersmann et al. (2019) and Fiorentino and Fund-Reznicek (2009) findings. Although priming was significant across all three item types (see top three rows in Table 6), including the orthographic control condition, the significantly larger magnitude of priming in the two compound conditions suggests that the here reported compound priming effects were not just simply due to orthographic prime-target overlap. This is in line with Gonzalez-Alonso et al. (2016), Fiorentino & Fund-Reznicek (2009), and J. Li et al. (2017) as well as a substantial number of studies comparing the size of masked priming effects in truly-affixed and pseudo-affixed word stimuli (e.g., Beyersmann et al., 2011, 2016, 2019; Longtin et al., 2003; Marslen-Wilson et al., 2008; Rastle et al., 2000, 2004; Rastle & Davis, 2008). The absence of a difference between the transparent (*snowball-SNOW*) and opaque (*honeymoon-HONEY*) conditions suggests that L1 readers rapidly and automatically decompose morphologically complex words into their morphemic subunits, independently of semantics. The automaticity of opaque compound priming in L1 challenges distributional theories of complex word processing (e.g., Feldman, 2000; Marelli et al., 2020) by which it is assumed that semantically transparent words produce more priming than semantically opaque words, a finding that was not confirmed in the current study. Instead, the results appear to bolster the notion that the early stages of complex word recognition are semantically blind, which is consistent with the assumptions of decompositional accounts (e.g., Beyersmann et al., 2011; Grainger & Beyersmann, 2017; Rastle et al., 2004; Taft, 2004; Taft, 2023). For example, the Activation Using Structurally Tiered Representations and Lemmas (AUSTRAL) model by Taft (2023) proposes that masked priming for *snowball-SNOW* arises from the activation via the lemmas of its individual constituents (e.g., the lemma for *snowball* is activated via the lemmas for *snow* and *ball*, the latter having been activated from the form units *snow* and *ball*). The lemma for *honeymoon* is activated directly from the form units for *honey* and *moon*, but the lemmas for *honey* and *moon* are also activated, hence leading to *honeymoon-HONEY* priming. On the other hand, the *wich* of *sandwich* has no lemma representation, which explains the here reported reduced magnitude of priming in the *sandwich-SAND* condition.

Experiment 1B revealed comparable transparent, opaque, and form priming effects in L2 English late but proficient bilinguals (see Table 6). This finding suggests that compound word processing in L2 is sensitive to form-based processing, consistent with prior observations from affixed words (e.g., Ciaccio & Jacob, 2019; Viviani & Crepaldi, 2022). We note that although this pattern differed from the pattern of L1 priming in Experiment 1A, the combined L1 and L2 analyses did not reveal a significant three-way interaction between relatedness, prime type, and group, suggesting that L2 processing shows a similar pattern to L1 processing. Theoretical implications of the here observed differences and similarities between L1 and L2 morphological priming are discussed further below.

**Figure 2:**
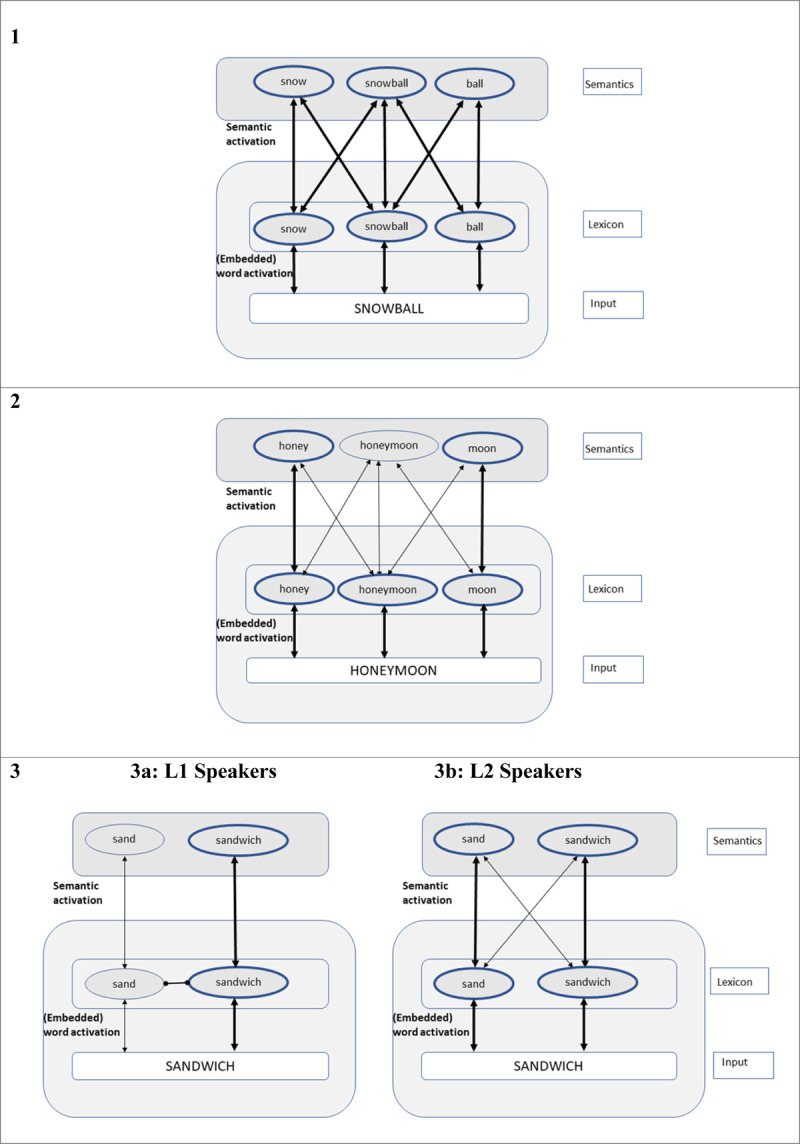
Visual Recognition of Compound Words in Monolinguals and Bilinguals. *Note*: Embedded constituents are extracted based on the stem activation principles proposed within the word and affix model (Beyersmann & Grainger, 2023; Grainger & Beyersmann, 2017). Thicker arrows and nodes represent greater levels of activation arising from the degree of relatedness in meaning between the constituents and the whole word. Panels 1 and 2 depict the processing of transparent (snowball) and opaque compound words (honeymoon), which is comparable across L1 and L2. Panel 3 represents the processing of non-morphological form controls (sandwich), with stronger form priming in L2 (panel 3b) than in L1 (panel 3a).

Particularly relevant within the context of the current study is a recent decompositional approach to complex word processing in bilinguals (Kahraman & Beyersmann, 2023). Building on the assumptions of the word and affix model (Beyersmann & Grainger, 2023), as well as the idea that the languages of bilinguals are co-activated in a language non-selective manner during visual word identification (see Multilink model, Dijkstra et al., 2019; BIA+ model, Dijkstra & van Heuven, 2002), it is proposed that orthographic input (in the form of position-coded letter identities; Grainger & Beyersmann, 2017) does not only activate the lexical representations of its corresponding whole-word (e.g., *snowball, honeymoon*), but also the representations of words embedded at the left and right edges of a letter string (e.g., *snow, ball, honey, moon*; see Figure 2).

We hypothesise that in L1 speakers, the principle of morpho-orthographic full decomposition is used to assess whether the combined length of the embedded constituents (*snow* and *ball*) fully matches the length of initial input (*snowball*). In the case of a match (see Figure 2, panels 1 and 2 in which the combination of ‘*snow* and ‘*ball* as well as ‘*honey* and ‘*moon* forms a real compound word of ‘*snowball* and ‘*honeymoon*, respectively), the embedded constituents receive a boost of activation. However, in the form condition (Figure 2, panel 3a) where the length of active embedded word (e.g., *sand*) does not match up with the length of the input (i.e., *sandwich*), their activation is not boosted, resulting in slower target recognition times and hence smaller priming effects in this condition. Hence, similar to the predictions of the AUSTRAL model by Taft (2023), smaller priming in the form condition is purely due to the edge-aligned activation of the embedded words (e.g., *sand* in *sandwich*), where the remaining letters fail to contribute to a coherent morphological structure (as indicated by the oval arrow between ‘*sand*’ and ‘*sandwich*’). The edge-aligned embedded words in the transparent and opaque conditions (e.g., *snow, ball, honey, moon*) are initially activated, but their activation is enhanced since the remaining letters form a coherent compound word structure (*snowball, honeymoon*).

In L2 speakers, complex word processing is entirely based on lower-level morpheme activation and more susceptible to form overlap (Figure 2, panels 1, 2, and 3b). Any given letter string simultaneously activates all lexical representations via bidirectional (lexico-semantic) links between orthographic lexicon and semantic representations. For example, in a transparent visual stimulus ‘*snowball*’, its whole-word representation (*snowball*), its morphemic constituents (*snow, ball*) are activated simultaneously within the Chinese-English orthographic lexicon. Similarly, the semantically opaque stimulus ‘*honeymoon*’ activates its whole-word representation and morphemic constituents. In the case of a form stimulus ‘*sandwich*’ (Figure 2, panel 3b), its whole word representation and embedded stem (*sandwich, sand*) are activated. Since the early stages of visual word recognition is guided by the sub-lexical orthographic influences in L2 speakers, the morpho-orthographic decomposition check is bypassed, leading to faster reaction times and thus similar priming effects in this condition. One possibility is that the principle of morpho-orthographic full decomposition is entirely absent or not sufficiently skilled to modulate the early stages of complex word recognition in L2. Alternatively, it is also possible that the depth of language processing and/or semantic analysis combined with the short prime duration are more restricted in the L2 speakers, limiting their ability to perform a rapid morphological and semantic analysis of the input letter string. Lexical representations are proposed to be fuzzier and less precise in L2 readers (e.g., Bordag et al., 2022; Gor et al., 2021), providing a framework for understanding a range of phenomena observed in the current L2 data, including slower lexical access and weaker lexical competition between active lexical representations. The semantics of compounds might appear *fuzzy* due to the interaction of multiple free fuzzy morphemes (e.g., Gor et al., 2021). A central factor contributing to the fuzziness of L2 lexical representations is the fact that the L2 lexicon (in unbalanced late bilinguals) develops when the L1 lexicon is already acquired and hence established, leading to less precise and weaker L2 than L1 lexical representations. Hence, this may explain the reported absence of a difference between compound and noncompound word priming effects in this speaker group.

### Individual Differences in L2 Form Priming

Priming effects in L2 were modulated by individual differences in L2 proficiency and exposure (Experiment 1B), showing that form priming decreased with increasing language proficiency and disappeared in the high proficiency group (for converging evidence, see Andrews & Hersch, 2010; Andrews & Lo, 2012, 2013; Viviani & Crepaldi, 2022).

These findings support the Lexical Quality Hypothesis (Perfetti, 1992; Perfetti & Hart, 2002) that priming effects from tasks that involve word identification (e.g., lexical decision task) are influenced by the degree of variation in the quality of lexical representations. As language proficiency and the quality of lexical representations increases, the connections between phonological, orthographic, and semantic information become tighter and increasingly automatised. We hypothesise that in the case of participants with lower proficiency, imprecise lexical representations lead to greater reliance on orthographic knowledge, thereby resulting in reduced levels of lexical inhibition from other competing candidate representations, which may in turn explain the facilitatory form priming effects in these individuals (Andrews & Hersch, 2010). In contrast, the larger magnitude of morphological priming in readers with higher proficiency, as evidenced in Experiment 1B, suggests that general language proficiency along with the degree of L2 exposure is an indicator for readers’ ability to detect morphemic structure (as also previously argued by Andrews & Lo, 2013).

### Future Directions

One interesting point to note for future research is that items in the orthographic control condition may need to be more carefully controlled to potentially tease apart the different influences of morphological versus form priming effects. It is worth remembering that although the present study revealed significant morphological priming effects in L1 speakers (Experiment 1A) but not in L2 speakers (Experiment 1B), the combined analyses across experiments did not yield a significant three-way interaction between speaker groups and priming effects. This was likely due to the large form priming effects across speaker groups, but nevertheless raises questions regarding differences in morphological priming between L1 and L2 speakers, which may have to be further evaluated in future, larger-scale bilingual priming studies. Relatedly, although form priming effects in L1 speakers were significantly smaller than the transparent and opaque compound priming effects, it suggests that some degree of facilitation was also present for prime-target pairs sharing an orthographic relationship only. This is not entirely consistent with the findings of previous work. While some studies have reported facilitatory form priming effects (Forster, 1987, p. 197; Forster & Veres, 1998), others have shown an inhibitory trend in this condition (e.g., Beyersmann et al., 2016, 2019; Grainger & Beyersmann, 2017; Viviani & Crepaldi, 2022). Davis and Lupker (2006) explored some possibilities that might explain the discrepant results in form priming effects (i.e., some studies have produced facilitation, whereas some others have observed inhibition from real word primes). These include that (1) the lower the prime word frequency relative to the target word frequency, the less inhibition, (2) the less difficult the lexical decision task based on nonword trials, the less inhibition, (3) the less the prime is to activate neighbours of the target, the less inhibition. In line with Andrews and Hersch (2010) and Andrews and Lo (2012), the present findings point to another issue that could potentially explain these empirical inconsistency: individual differences in vocabulary knowledge.

A further potential avenue for future research is the exploration of how L1 translation equivalents influence compound processing in L1 Chinese – L2 English bilinguals. Previous studies have shown that Chinese translations are activated when L2 speakers read English words (e.g., Thierry & Wu, 2007; Wen & van Heuven, 2018; Zhang et al., 2011). This may shed light on the cognitive processes underlying compound word recognition in L2 speakers of English with Chinese as their L1. Future work could use a priming paradigm that requires L1 activation in L2 reading and examine whether this co-activation leads to stronger priming effects for transparent, opaque, and form conditions compared to L1 speakers.

## Conclusion

The current study used the masked primed lexical decision paradigm to examine orthographic and semantic influences on compound word processing in English monolinguals and Chinese – English late bilinguals. By building on the important methodological and theoretical foundations of Jonathan Grainger and colleagues, we showcase the strength of the masked priming paradigm in teasing apart fine-tuned differences in orthographic, morphological, and semantic similarity. While late bilinguals appear to be primarily susceptible to L2 orthographic prime-target overlap, L1 speakers rely on a form of rapid, semantically independent morphological analysis. This supports the idea that automatic morphological segmentation requires a relatively high level of language expertise.

## Data Accessibility Statement

Experiments 1A and 1B were preregistered prior to conducting the research. The materials, the raw data, and analysis script are available at https://osf.io/cqkw3/?view_only=688e8ce2d84b4088b357e7ba1a5b4192.

## Footnotes

J. Li et al. (2017) found a significant form priming effect for L2 speakers with lower proficiency (Group C), which was comparable in size to the transparent priming effect. However, note that this effect was absent for L2 speakers with higher proficiency (Group I), who only showed a significant transparent priming effect. Thus, the existence of L2 morphological priming might be contingent on the proficiency level of the L2 speakers.

Number of years of formal education and number of years spent in an English-speaking country.

Age when participants began acquiring English.

Age when participants began reading in English.

Participants rated their proficiency on a rating scale from 0–10 on the domains of speaking, understanding spoken English, reading in English where 0 and 10 denoted to “none” and “perfect”, respectively.

Participants rated to which extent they were exposed to English on rating scale from 0–10 in interacting with friends and family, watching TV and listening to radio/music, reading, and language-lab/self-instruction. 0 and 10 denoted to “none” and “always”, respectively.

Age at which participants became fluent in English. We believe that this definition is more realistic given that participants were not immersed in the foreign language at the time of the testing.

Based on how much of a foreign accent a participant has in English in their own perception.

Based on how frequently others identify participants as a non-native speaker based on their accent in English.

Note that the Windows 10 operating system (OS) was uniformly used on both the lab computers and the personal computers of all participants, with the exception of one individual that used an earlier version Windows OS.

It cannot entirely be ruled out that individuals who participated online used a dictionary or other aids to proceed through the testing, as is true for all online testing in general. However, the use of external aids in this study is rather unlikely, because participants had to complete speeded response tasks primarily, and since they were not marked or graded for their performance in the task, they had little incentive of achieving top results in the individual proficiency tasks.

The final model was: –1000/rt ~ relatedness * primeType * modality + scale(trial)+ scale(primefreq)+ scale(primeOrthN)+ scale(primePhonN)+ (relatedness + modality + primeType|ID) + (relatedness + modality + scale(trial) +scale(primeOrthN)|target), dataL1,control=lmerControl(optimizer=”bobyqa”, optCtrl=list (maxfun=2e5),calc.derivs=F)).

To gain a more nuanced understanding of the impact of individual differences in L2 participants’ language background and experience on priming data, participants completed the Language Experience and Proficiency Questionnaire (LEAP-Q, Marian et al., 2007). The questionnaire included linguistic variables such as self-reported language proficiency and exposure, as well as age of language acquisition.

The impact of each test in the reading skills battery was computed in a separate model to avoid collinearity. One example of lmer syntax (in this case, for the vocabulary test) is as follows: –1000/rt ~ relatedness * primeType * Vocab.z +scale(trial)+ scale(primefreq)+ scale(primeOrthN)+ scale(primePhonN)+ (relatedness|subj) + (relatedness|target), dataL1, REML = T.

To calculate the proficiency test scores for in-person and web-delivered testing, we determined the proportion of correct responses (e.g., in-person: the number of correct responses divided by 16; web-delivered: the number of correct responses divided by 60 for the vocabulary test). We then created separate z-scores for the in-person and web-delivered data.

Scores of English language proficiency and exposure from LEAP-Q were correlated and hence summed to create a composite measure of ‘English proficiency/exposure’ in line with prior research (e.g., Freeman et al., 2022).

One reviewer suggested that more proficient L2 speakers might show L1-like results and therefore including them in the combined data of L1 and L2 groups might have diluted the results. Based on this suggestion, we excluded the top quartile of L2 speakers using individual language proficiency+ L2 exposure scores (n = 20) and re-run analyses. The pattern of results did not change. However, there was a statistically significant group difference between Form and Transparent conditions (*β* = –0.04, SE = 0.02, z = –2.01, p = .04), which is due to morphological facilitation independently of form overlap in L1.
